# Extensive Small Bowel Diverticulosis Incidentally Discovered During Surgery for Small Bowel Volvulus

**DOI:** 10.7759/cureus.105038

**Published:** 2026-03-11

**Authors:** Rachel Rayden, Lucas Marques, Robin Berk

**Affiliations:** 1 Surgery, Albert Einstein College of Medicine, New York, USA; 2 Psychiatry, Albert Einstein College of Medicine, New York, USA; 3 General Surgery, Montefiore Medical Center, New York, USA

**Keywords:** small bowel diverticulitis, small bowel diverticulosis, small-bowel volvulus, surgery general, upper gastrointestinal surgery

## Abstract

Small bowel diverticulosis is a rare condition that is often asymptomatic and discovered incidentally. We present a case of extensive small bowel diverticulosis in a woman in her early 90s who was found to have numerous large diverticula during surgery for small bowel volvulus secondary to an adhesive band, requiring segmental small bowel resection. Although the diverticulosis was an incidental finding and was unlikely to have caused the volvulus and subsequent bowel ischemia, small bowel diverticulosis can present with serious complications. Its pathogenesis remains poorly understood. This case highlights the importance of considering small bowel diverticulosis in elderly patients presenting with nonspecific abdominal symptoms or bowel obstruction, as the condition may not be apparent on imaging and may only be identified intraoperatively.

## Introduction

Non-Meckel's small bowel diverticulosis has a reported prevalence of 0.3% to 2.3% in the general population [1]. Approximately 15% of cases occur in the jejunum, 15% in the ileum, and 5% affect both segments [2]. Unlike colonic diverticulosis, it is often asymptomatic and is typically discovered incidentally during imaging, surgery, or endoscopy [3]. When symptoms do occur, they are usually nonspecific, such as intermittent abdominal discomfort, malabsorption, or gastrointestinal bleeding. Complaints such as intermittent abdominal pain, constipation, and diarrhea have been reported in up to 90% of patients presenting with symptomatic small bowel diverticulosis [4]. Common complications include diverticulitis, perforation, and obstruction [5]. Jejunoileal diverticulitis is an uncommon complication, with a mortality rate reported to be as high as 24% [6].

The etiology of small bowel diverticulosis is not yet fully understood but is believed to involve abnormalities in intestinal motility and increased intraluminal pressure, leading to the herniation of mucosa and submucosa through points of weakness in the muscular layer of the small bowel [7]. Due to the vague clinical presentation and low prevalence of the condition, small bowel diverticulosis remains difficult to diagnose. In the absence of complications, it is often misdiagnosed, or its diagnosis is delayed [8,9]. Here, we describe a rare case of extensive small bowel diverticulosis in a female patient in her early 90s, detailing its presentation, clinical features, diagnosis, and management.

## Case presentation

A woman in her early 90s presented to the emergency department with five days of abdominal distension, decreased appetite, and intolerance to oral intake. She reported no vomiting, fever, chills, or weight loss and had regular bowel movements with passage of flatus.

Her past medical history included asthma, carpal tunnel syndrome, hypertension, hyperlipidemia, type II diabetes mellitus, stage three chronic kidney disease, and Pseudomonas bacteremia in 2019. Her past surgical history included cholecystectomy and open total abdominal hysterectomy with bilateral salpingo-oophorectomy. Small bowel diverticulosis was not visualized during either procedure, suggesting that the condition was acquired. A diagnostic colonoscopy performed after an episode of rectal bleeding in 2017 revealed internal hemorrhoids, a 4 mm polyp in the ascending colon, and colonic diverticulosis.

On presentation, the patient was afebrile, hemodynamically stable, and in no acute distress. On examination, her abdomen was markedly distended and tympanic to percussion, without tenderness or signs of peritonitis (Table 1).

**Table 1:** Significant laboratory studies

Laboratory study	Value	Reference range
White blood cell count	9.4×10³/µL	(4.5-11.0)×10³/µL
Hemoglobin	13.1 g/dL	14-18 g/dL (men); 12-16 g/dL (women)
Platelet count	423×10³/µL	(150-350)×10³/µL
Sodium	148 mmol/L	136-142 mmol/L
Blood urea nitrogen	160 mg/dL	8-23 mg/dL
Creatinine	4.96 mg/dL	0.6-1.2 mg/dL

Investigation 

**Figure 1:**
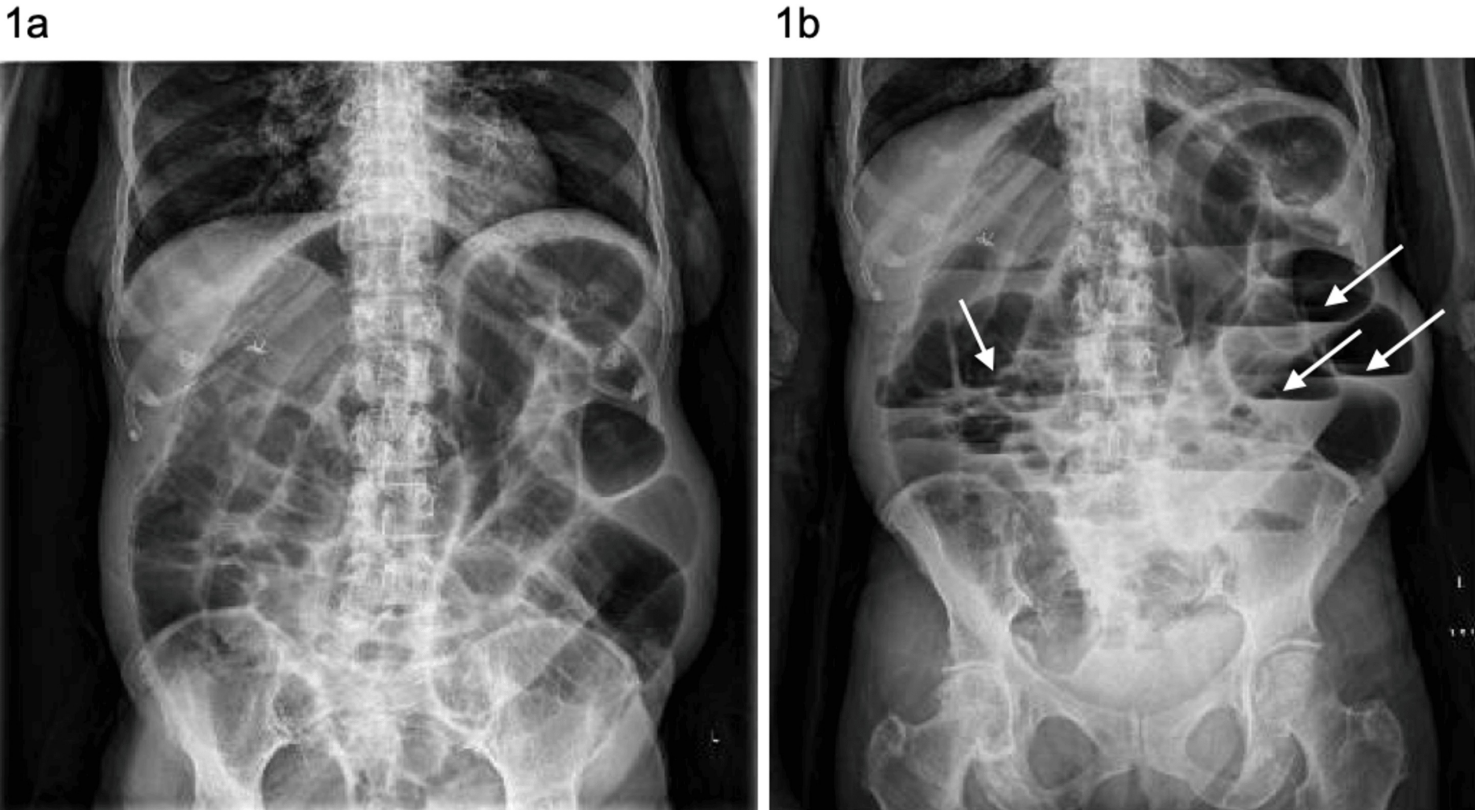
Supine (1a) and upright (1b) abdominal X-rays illustrating severe bowel distension Severe small bowel dilatation with air-fluid levels, indicated by arrows, was visualized on abdominal X-ray, supporting the diagnosis of small bowel obstruction and justifying surgical exploration. Large, diffuse small bowel diverticula were not visible on the X-ray and were unexpectedly discovered during surgery. Although clinically silent, this finding demonstrates widespread small bowel diverticulosis.

**Figure 2:**
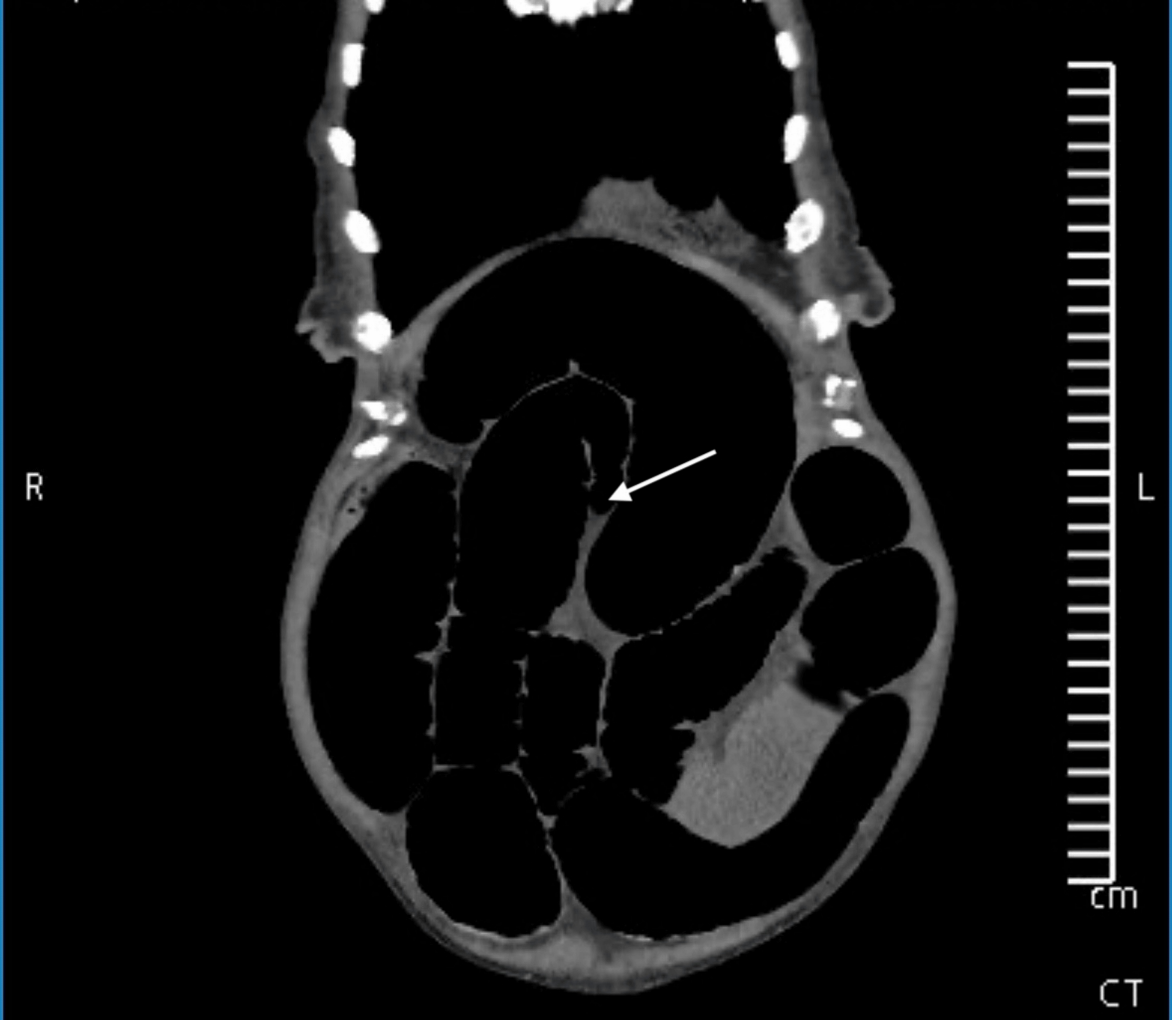
Coronal non-contrast CT image showing a small bowel transition point A non-contrast CT scan revealed dilated loops of small bowel (up to 5.6 cm) and mesenteric torsion around a transition point, indicated by an arrow. The patient's extensive small bowel diverticulosis was not visualized on imaging. This highlights that small bowel diverticulosis is often not detected radiologically, particularly in non-contrast or emergent settings, such as in this case. Small bowel diverticulosis is rare (0.3%-2.3% incidence) and is typically clinically silent. It is often identified incidentally during surgery. R: right; L: left

An abdominal X-ray revealed dilated loops of bowel (Figure 1). CT imaging revealed small bowel dilatation measuring up to 5.6 cm, with twisting of the bowel and mesentery at a transition point in the mid-abdomen (Figure 2). Significant colonic diverticulosis was also noted. The findings were concerning for small bowel obstruction secondary to an internal hernia or volvulus.

Treatment 

**Figure 3:**
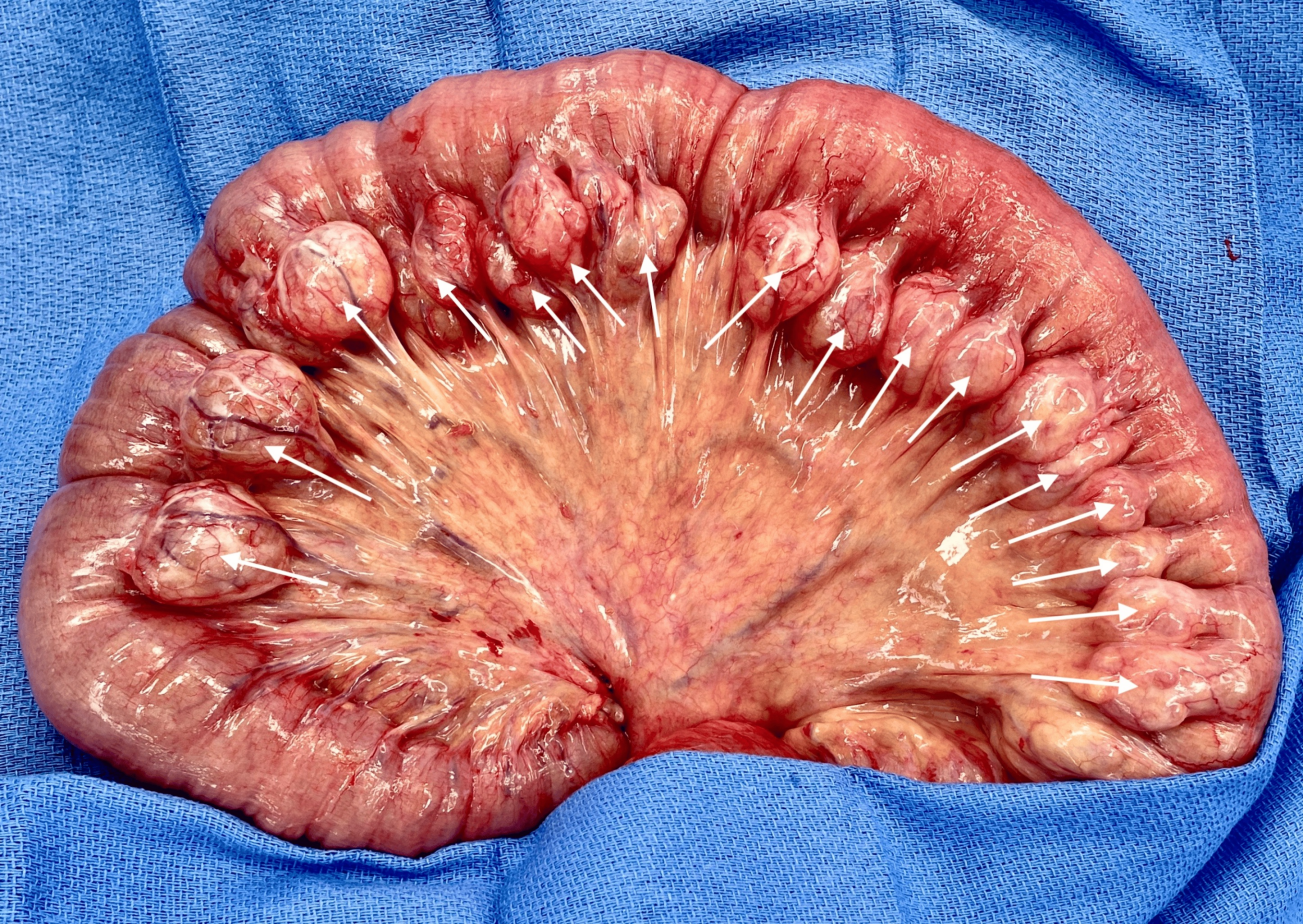
Intraoperative appearance of diffuse small bowel diverticulosis Exploratory laparotomy demonstrating small bowel volvulus secondary to an adhesive band, requiring resection of ischemic bowel. Numerous large small bowel diverticula were discovered incidentally.

Based on these findings, the patient was taken to the operating room for diagnostic laparoscopy. Upon entry into the abdomen, the small bowel was found to be volvulized around an adhesive band. The bowel was significantly dilated, which obstructed laparoscopic visualization; therefore, the procedure was converted to an open laparotomy for better visualization and careful handling of the friable bowel. Inspection of the small bowel revealed numerous large diverticula distributed throughout its entire length (Figure 3). The adhesive band was lysed, and a 10 cm segment of nonviable small bowel, approximately 10 cm from the terminal ileum, was resected due to ischemia. Because the patient was unstable and required vasopressor support during the index operation, the bowel was left in discontinuity, and a temporary abdominal closure was placed to allow rapid transfer to the ICU for resuscitation. Given the ongoing vasopressor requirement and questionable bowel viability, the abdomen was left open with temporary closure for planned re-exploration. During a second-look operation two days later, a completion ileocecectomy with primary anastomosis and abdominal fascial closure was performed.

Outcome and follow-up 

Two days later, the patient was taken back to the operating room for a second-look operation, during which an ileocecectomy with primary anastomosis and abdominal fascial closure was performed. One week after her second surgery, she was found to have a pelvic hematoma with active extravasation on imaging and was subsequently taken for an exploratory laparotomy with evacuation of the hematoma.

Three weeks later, the patient was readmitted with abdominal pain, diarrhea, and coffee-ground emesis. Non-contrast CT of the abdomen and pelvis revealed mildly distended small bowel without a transition point and wall thickening of the left and sigmoid colon. She was found to have significant leukocytosis and worsening renal function. Stool studies were positive for *Clostridioides difficile* colitis, and she was started on oral and rectal vancomycin and Zosyn. 

Given the patient’s worsening renal function, leukocytosis, and lactic acidosis, she was transferred to the surgical intensive care unit (SICU) for presumed septic shock. After goals-of-care discussions with the family, the decision was made to proceed with comfort care. The patient was designated do-not-resuscitate and do-not-intubate and ultimately passed away.

## Discussion

Small bowel diverticulosis is an uncommon condition that is often discovered incidentally during imaging, endoscopy, or surgical procedures [1-3]. In contrast to colonic diverticulosis, it is rare and typically asymptomatic, with an estimated prevalence of 0.3% to 2.3% in the general population that increases with age [1,2]. The diverticula usually arise along the mesenteric border of the small intestine, where blood vessels penetrate the muscularis propria and create areas of weakness. These lesions are pseudodiverticula, consisting of mucosa and submucosa that herniate through the muscular layer [4]. The pathophysiology remains incompletely understood but may involve disordered intestinal motility, elevated intraluminal pressures, and focal wall weakness [7]. When symptoms occur, they are often nonspecific and may include intermittent abdominal pain, bloating, malabsorption, diarrhea, or constipation [4].

Complications, though uncommon, can be serious and include diverticulitis, bleeding, perforation, volvulus, and obstruction [5,6]. Jejunoileal diverticulitis carries a mortality rate of up to 24%, underscoring the importance of early recognition in symptomatic cases [6]. Although our patient’s diverticulosis was not the cause of her obstruction, prior reports describe cases in which small bowel diverticula have resulted in acute obstruction or perforation requiring urgent surgical intervention, particularly in older adults [10-13].

Diagnosis is frequently missed radiologically. In our patient, preoperative CT imaging demonstrated small bowel obstruction with a transition point and colonic diverticulosis, but did not identify small bowel diverticulosis. This is consistent with existing literature showing that small bowel diverticula are often overlooked, especially on acute or non-contrast scans [7-9]. The small bowel’s length and convoluted anatomy, combined with the thin-walled and non-inflamed nature of uncomplicated diverticula, make them difficult to detect. In contrast, complications such as diverticulitis, perforation, or abscess formation are more readily visualized due to associated wall thickening or fat stranding [9].

Management is guided by the presence and severity of complications. Asymptomatic diverticulosis requires no treatment or routine surveillance, whereas symptomatic but uncomplicated cases may be managed conservatively [12]. A complicated disease, including perforation, abscess, hemorrhage, or obstruction, warrants urgent intervention such as percutaneous drainage or segmental resection, depending on clinical stability [14-16]. Awareness of this spectrum is particularly important in elderly patients, in whom delayed recognition may significantly worsen outcomes.

This case demonstrates a striking example of extensive small bowel diverticulosis incidentally discovered during surgery for adhesive small bowel volvulus. The diverticulosis itself was not the source of obstruction, yet its diffuse nature highlights the variability of disease burden. Intraoperative inspection remains essential, as small bowel diverticulosis may not be detected preoperatively, particularly in emergent settings.

## Conclusions

A woman in her early 90s was found to have extensive small bowel diverticulosis during surgery for a small bowel volvulus requiring resection and ileocecectomy. Her postoperative course was complicated by a pelvic hematoma, renal failure, and septic shock. Although the diverticulosis was an incidental finding and unlikely to have contributed to the volvulus, it remains clinically significant because small bowel diverticulosis, though rare, can present with life-threatening complications in elderly patients.
